# Implementation of a cystic fibrosis lung transplant referral patient decision aid in routine clinical practice: an observational study

**DOI:** 10.1186/s13012-015-0206-4

**Published:** 2015-02-07

**Authors:** Dawn Stacey, Katherine L Vandemheen, Rosamund Hennessey, Tracy Gooyers, Ena Gaudet, Ranjeeta Mallick, Josette Salgado, Andreas Freitag, Yves Berthiaume, Neil Brown, Shawn D Aaron

**Affiliations:** Ottawa Hospital Research Institute, Faculty of Health Sciences, University of Ottawa, 451 Smyth Road, Ottawa, ON K1H 8M5 Canada; Ottawa Hospital Research Institute, 501 Smyth Road, Ottawa, ON K1H 4E9 Canada; McMaster University, 1200 Main St. Hamilton, Hamilton, ON L8N 2Z5 Canada; University of Western Ontario, 800 Commissioner’s Rd. E, London, ON N6A 4G5 Canada; The Ottawa Hospital, 501 Smyth Road, Ottawa, ON K1H 4E9 Canada; University of Alberta, 8440-112 Street, Edmonton, AB T6G 2B7 Canada; Institut de recherches cliniques de Montréal, 110 avenue des Pins Ouest, Montréal, QC H2W1R7 Canada; Ottawa Hospital Research Institute, Faculty of Medicine, University of Ottawa, 451 Smyth Road, Ottawa, ON Canada

**Keywords:** Implementation, Cystic fibrosis, Patient decision aid, Lung transplantation, Decision support, Barriers

## Abstract

**Background:**

The decision to have lung transplantation as treatment for end-stage lung disease from cystic fibrosis (CF) has benefits and serious risks. Although patient decision aids are effective interventions for helping patients reach a quality decision, little is known about implementing them in clinical practice. Our study evaluated a sustainable approach for implementing a patient decision aid for adults with CF considering referral for lung transplantation.

**Methods:**

A prospective pragmatic observational study was guided by the Knowledge-to-Action Framework. Healthcare professionals in all 23 Canadian CF clinics were eligible. We surveyed participants regarding perceived barriers and facilitators to patient decision aid use. Interventions tailored to address modifiable identified barriers included training, access to decision aids, and conference calls. The primary outcome was >80% use of the decision aid in year 2.

**Results:**

Of 23 adult CF clinics, 18 participated (78.2%) and 13 had healthcare professionals attend training. Baseline barriers were healthcare professionals’ inadequate knowledge for supporting patients making decisions (55%), clarifying patients’ values for outcomes of options (58%), and helping patients handle conflicting views of others (71%). Other barriers were lack of time (52%) and needing to change how transplantation is discussed (42%). Baseline facilitators were healthcare professionals feeling comfortable discussing bad transplantation outcomes (74%), agreeing the decision aid would be easy to experiment with (71%) and use in the CF clinic (87%), and agreeing that using the decision aid would not require reorganization of the CF clinic (90%). After implementing the decision aid with interventions tailored to the barriers, decision aid use increased from 29% at baseline to 85% during year 1 and 92% in year 2 (*p* < 0.001). Compared to baseline, more healthcare professionals at the end of the study were confident in supporting decision-making (*p* = 0.03) but continued to feel inadequate ability with supporting patients to handle conflicting views (*p* = 0.01).

**Conclusion:**

Most Canadian CF clinics agreed to participate in the study. Interventions were used to target identified modifiable barriers to using the patient decision aid in routine CF clinical practice. CF clinics reported using it with almost all patients in the second year.

## Background

Cystic fibrosis (CF) is one of the most common inherited fatal diseases [1]. Although survival has improved dramatically over the last 50 years, many patients with CF develop end-stage lung disease in young adulthood or middle age [1]. For patients with end-stage lung disease, lung transplantation can improve exercise tolerance, quality of life, and survival [2]. However, there are significant risks or inconveniences with lung transplantation including infection, implant rejection, survival beyond 5 years limited to 50%–60%, relocation to only one of five transplant centers in Canada, and need to build a relationship with a CF transplantation healthcare team [3].

Adults with CF experience considerable difficulty making the decision about lung transplantation [4,5]. To help these patients, we previously developed the CF lung transplant referral patient decision aid based on the Ottawa Decision Support Framework and the International Patient Decision Aid Standards [6,7]. Elements in the decision aid include focus on an explicit decision, best available evidence on treatment options for end-stage CF lung disease (transplant versus supportive care), probabilities of benefits and risks, an explicit values-clarification exercise, and structured guidance in making the decision. This patient decision aid includes a one-page summary report to facilitate sharing the patients’ knowledge, values, and preferences with healthcare professionals. This one-page report can be filed on the patients’ health record [8].

We conducted a randomized controlled trial to evaluate this CF lung transplantation referral patient decision aid at nine CF clinics in Canada and five in Australia [9]. Compared to usual care, patients randomized to the patient decision aid had greater knowledge, more realistic expectations about the benefits and risks of lung transplantation, lower decisional conflict, and higher satisfaction with the decision-making process. Our findings were consistent with other trials of patient decision aids [10]. Our findings were disseminated in presentations at conferences, journal publications, and by adding the patient decision aid (English, French) to the international A to Z patient decision aids inventory [http://decisionaid.ohri.ca/decaids.html] [4,9,11,12].

Despite positive patient outcomes from using the patient decision aid and the use of passive dissemination of the study findings, there was no evidence indicating that it was routinely being used in adult CF clinics. Common barriers consistently interfering with implementation of patient decision aids across multiple studies were that healthcare professionals: had inadequate training regarding their use, were indifferent about using it, lacked confidence in the patient decision aid content, and were concerned about disrupting established workflows [13]. Another systematic review showed that healthcare professional training increased shared decision-making and use of patient decision aids [14]. Importantly, research has shown that successful implementation of evidence into clinical practice requires targeted and tailored interventions based on identified barriers to changing healthcare professionals’ behaviors [15,16].

The purpose of this study was to evaluate a sustainable approach for implementing the lung transplant referral patient decision aid into clinical practice in adult CF clinics. Specific objectives were: a) to monitor the change in use of the CF lung transplant referral patient decision aid after exposure to the interventions targeted to overcome identified barriers; and b) to assess change in healthcare professionals’ perceived barriers to using the patient decision aid with CF patients.

## Methods

A prospective pragmatic observational study was conducted from September 2010 to August 2012 and was guided by the Knowledge-to-Action Framework [17]. This framework is designed to enhance uptake of evidence-based innovations in clinical practice. After identifying an evidence-practice gap (i.e., CF lung transplant referral patient decision aid was not being used in clinical practice), next steps in the framework involve: a) assessing for adaptations, barriers, and facilitators to using the patient decision aid; b) choosing implementation interventions to overcome identified barriers; and c) monitoring use including sustained use of the patient decision aid. Sustainability is most often measured 2 years or more after initial implementation to determine continued use after initial efforts to increase adoption [18,19]. Our study was approved by The Ottawa Hospital Research Ethics Board.

### Setting and participants

All 23 accredited adult CF clinics within eight different provincial healthcare systems in Canada were eligible to participate. Patients with CF attend these clinics routinely (e.g., every 3 to 4 months) and have their lung function measured at each clinic visit. Most CF clinics have one specialized physician and one nurse coordinator. Usually, both are responsible for counseling patients making decisions about referral for lung transplantation. Other healthcare professionals such as pharmacists and social workers may be involved in some clinics. Healthcare professionals who routinely counseled patients from these 23 clinics were invited to participate in our study. To increase awareness of the study, a 1-h presentation on shared decision-making was provided to Canadian CF healthcare professionals at the North American CF Conference in October 2010.

### Surveys

**Figure 1:**
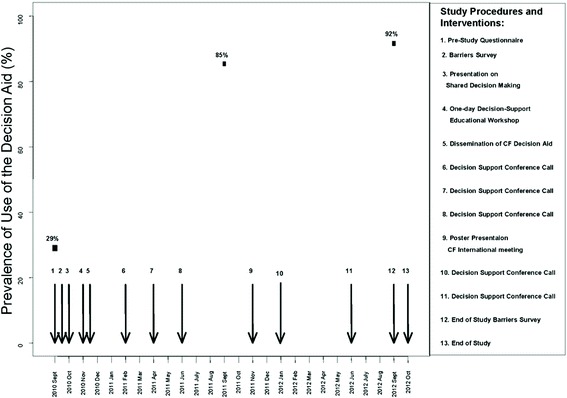
**Interventions and timeline illustrating change in use of the patient decision aid over the 2-year study period (** ***n*** **= 15 CF clinics).** Each arrow indicates the timing of a separate study procedure or intervention.

We conducted two types of surveys: patient decision aid use and barriers assessment (see Figure 1). At the start of the study, CF clinics were emailed to determine the number of patients in their clinic during the year prior to study initiation (September 2009–August 2010) who had: a) engaged in a lung transplant referral discussion; b) received a lung transplant referral; and c) received the patient decision aid. Whether or not healthcare professionals participated in the study interventions, participating CF clinics were re-surveyed from September 2011 to August 2012 to determine these same outcomes. At participating clinics, tracking logs were used to monitor all potential referrals to transplant centers and whether or not the CF lung transplant referral patient decision aid was used. CF nurse coordinators sent the tracking logs to the study coordinator (KV) when referral for lung transplantation was discussed with a new patient. The tracking log included date of the discussion, use of the patient decision aid (yes/no), timing of patient decision aid use, date of follow-up discussion, and any general comments. This survey and the tracking logs provided data on the monitoring use phase of the Knowledge-to-Action Framework.

The barriers assessment survey and a copy of the patient decision aid were sent to nurse coordinators and CF physicians at the start of the study (September–November 2010). The ‘barriers survey’ measured healthcare professionals’ perceived barriers and facilitators towards using the patient decision aid using five items at the level of the healthcare professionals (e.g., knowledge, confidence, skills) and six items at the level of the organization (e.g. adequate time, fit with workflow, ease of use in practice). Respondents were instructed to rate each statement on a 5-point Likert scale ranging from 1 (strongly disagree) to 5 (strongly agree) with neutral in the center. The survey items were previously validated in a study of physicians using principal component analysis [20] and subsequently validated in studies of nurses and other non-physician healthcare professionals [21,22]. At the end of the study, the survey was re-administered to those who responded to the baseline survey (July–August 2012). The barriers assessment survey was conducted to be consistent with the Knowledge-to-Action Framework, and previous studies were more successful with implementing shared decision-making when barriers were assessed [14].

### Implementation interventions

Results from the barriers survey and evidence on effective interventions were used to tailor the interventions for implementing the patient decision aid into routine clinical practice [23]. Table 1 maps interventions onto the barriers and Figure 1 presents the timeline schematic for delivery of these interventions.

**Table 1:** **Multifaceted interventions used to overcome modifiable barriers**

**Barriers**	**Implementation interventions**			
	**Educational workshop (5 h)**	**Online tutorial (1.5 h)**	**Easy access to the patient decision aid**	**Conference calls for ongoing support**
Need knowledge about supporting patients in health decisions	X	X	X	X
Lack of confidence in ability to support patients	X			X
Need for skills in helping patients clarify their values	X		X	
Inadequate time in clinic to discuss decisions with patients	X		X	X
Need to enhance ability to support patients handling conflicting views	X	X		X
Perception that using the PtDA will require major changes in current practice	X			X

A *five-hour workshop* was provided to increase healthcare professionals’ knowledge and skills in using patient decision aids and addressed several of the barriers. An expert in patient decision aids and training healthcare professionals facilitated the workshop (DS). The workshop objectives are available at https://decisionaid.ohri.ca/training.html. Two role play scenarios were used by all participants to build skills and increase their confidence in using the patient decision aid with patients. Informal performance feedback was provided within the role playing exercises using the Decision Support Analysis Tool [24]. The workshop was free of charge, but participants were reimbursed for travel costs and received an honorarium ($200 Canadian). At the end of the workshop, participants were asked to complete a knowledge test and satisfaction survey. The knowledge test administered post-workshop was the same ten questions used in the online tutorial [25]. Consistent with other training programs, the satisfaction survey aimed at assessing participants’ overall rating as well as whether or not the workshop achieved the learning objectives and provided adequate information, new information, and enough role play exercises [26]. A similar workshop was shown to improved healthcare professionals’ knowledge and skills compared to a control group [27,28].

The *Ottawa Decision Support Tutorial* in English and French was offered to healthcare professionals who were unable to attend the workshop [25]. This online tutorial provides a series of ten modules with review questions for each module. It takes about 90 min to complete. The tutorial has similar knowledge content to the workshop described above but did not include the skills building exercises or group discussions. The tutorial has been shown to improve healthcare professionals’ knowledge [27,28].

*Access to the patient decision aid* was enhanced by sending participating centers’ printed copies of the patient decision aid in English and French at the beginning of the study and anytime during the 2-year period when additional copies were requested. Online access to the patient decision aid was also provided [http://decisionaid.ohri.ca/decaids.html]. To facilitate implementation, use of the CF lung transplant referral patient decision aid was encouraged for patients with CF having a forced expiratory volume in one second (FEV_1_) of 30 to 40% predicted or when they experienced a rapid decline in lung function necessitating a hospital admission. The patient decision aid was implemented by having a healthcare professional ask patients to complete the patient decision aid on their own, discuss patients’ responses to the patient decision aid at the subsequent encounter, and collect the completed one-page summary report for inclusion on their clinic record. Given the content and structured decision-making guidance in the patient decision aid, it was also an intervention that could enhance healthcare professionals’ knowledge and skills in supporting patients facing this decision and change the way clinic time is used for discussing decisions [10].

*Conference calls* were used to reinforce learning and provide ongoing support. The calls were consistently structured by asking participants to share their positive and negative experiences with using the patient decision aid and discussing strategies to address implementation issues. Calls occurred every 3 months in the first year and every 6 months in the second year. During the calls, notes were taken to summarize the discussion. By encouraging participants to share their experiences using the patient decision aid, the calls were able to discuss several identified barriers (see Table 1).

### Statistical analysis

Characteristics of healthcare professionals were assessed using frequency distributions and univariate descriptive statistics. The primary outcome, sustained use of the patient decision aid by healthcare professionals for 80% of eligible patients at the end of year two, was analyzed using McNemar’s test to assess annual trends in use of the patient decision aid. Barrier survey responses were reclassified into agree (strongly agree, agree), disagree (strongly disagree, disagree) and neutral. This data was then analyzed using Friedman’s test taking into account that the baseline and post-intervention scores were not independent. All analyses were conducted using SAS software, version 9.1 (SAS Institute, Inc., Cary, North Carolina). Content analysis was used for qualitative survey feedback and conference call notes.

## Results

Of the 23 adult Canadian CF clinics, 18 agreed to participate, 3 agreed to provide data on the use of the patient decision aid only, and 2 clinics did not participate because they routinely referred all their CF patients with poor lung function for a lung transplant assessment without eliciting patients’ informed preferences. For the three who agreed to provide data on patient decision aid use, no rationale was given for not participating in the study interventions and they subsequently reported that the patient decision aid was not used with any of the 20 patients who were considering referral for lung transplantation.

### Baseline barriers survey

Thirty-one healthcare professionals completed the barriers survey with at least one from each of the 18 participating CF clinics. There were 18 nurses, 12 physicians, and 1 pharmacist, and their characteristics are presented in Table 2. The main barriers to using the patient decision aid in clinical practice were: a) healthcare professionals’ lack of knowledge and skills in supporting patients making decisions, clarifying values for outcomes of options, helping patients handle conflicting preferred options of others; b) lack of time, and c) needing to change how transplantation is discussed as a team (Tables 3 and 4). Facilitators at baseline were healthcare professionals feeling comfortable discussing bad transplantation outcomes, agreeing the patient decision aid would be easy to use in the CF clinic, and indicating that using it was unlikely to require reorganization of the CF clinic (Tables 3 and 4). Modifiable barriers are listed in Table 1 along with the multi-faceted interventions tailored to address these barriers.

**Table 2:** **Characteristics of healthcare professionals who completed the barrier survey**

**Characteristics**	**Participants (** ***n*** **= 31)**
*Age, median years (range)*	47 (26–65)
*Sex*	
Female	24 (77.4%)
Male	7 (22.6%)
*Type of CF healthcare professional*	
Nurses	18 (58%)
Physicians	12 (39%)
Pharmacist	1 (3%)
*Experience in their profession*	
<5 years	2 (6%)
6–10 years	3 (10%)
11–15 years	4 (13%)
16–20 years	3 (10%)
20–25 years	10 (32%)
>26 years	9 (29%)
*Experience in CF clinic*	
<12 months	4 (13%)
1–5 years	9 (29%)
6–10 years	3 (10%)
>10 years	15 (48%)

**Table 3:** **Healthcare professionals’ perceived barriers to patient decision aid use**

**Items**	**Pre (** ***n*** **= 31)**			**Post (** ***n*** **= 28)**			***p*** value*
	***Disagree***	***Neutral***	***Agree***	***Disagree***	***Neutral***	**Agree**	
Need to enhance my knowledge about supporting patients making health decisions	9	5	17	7	7	14	1.00
	(29%)	(16%)	(55%)	(25%)	(25%)	(50%)	
Feel confident in my ability to support patients making health decisions	1	4	26	0	1	27	0.03
	(3%)	(13%)	(84%)	(0%)	(4%)	(96%)	
Feel comfortable discussing bad outcomes for lung transplantation	4	4	23	0	4	24	0.10
	(13%)	(13%)	(74%)	(0%)	(14%)	(86%)	
Need to enhance my ability to support patients in handling conflicting views about the decision from others	4	5	22	4	10	14	0.01
	(13%)	(16%)	(71%)	(14%)	(36%)	(50%)	
Need to enhance my skills in helping patients clarify their values for option outcomes	6	7	18	7	9	12	0.08
	(19%)	(23%)	(58%)	(25%)	(32%)	(43%)	

*p value indicating change from baseline to end of study.

**Table 4:** **Organizational level barriers perceived to interfere with patient decision aid use**

**Items**	**Pre (** ***n*** **= 31)**			**Post (** ***n*** **= 28)**			***p*** value*
	***Disagree***	***Neutral***	***Agree***	***Disagree***	***Neutral***	**Agree**	
The decision aid will be easy to use in our CF program	1	3	27	1	2	25	0.56
	(3%)	(10%)	(87%)	(3%)	(7%)	(90%)	
There is adequate time in the clinic to discuss health decisions with patients	9	7	15	10	4	14	0.18
	(29%)	(23%)	(48%)	(36%)	(14%)	(50%)	
Using the decision aid will require reorganization of our CF program	20	8	3	22	3	3	0.53
	(64%)	(26%)	(10%)	(78%)	(11%)	(11%)	
Using the decision aid will not require major changes to the way we currently discuss the topic with our CF patients	6	7	18	2	4	22	0.36
	(19%)	(23%)	(58%)	(7%)	(14%)	(79%)	
The decision aid will be easy to experiment with before deciding to adopt it in our CF program	1	8	22	2	7	19	0.78
	(3%)	(26%)	(71%)	(7%)	(25%)	(68%)	
The decision aid is likely to be used by most of my colleagues	1	11	19	3	6	19	0.53
	(3%)	(35%)	(61%)	(11%)	(21%)	(68%)	

*p value indicating change from baseline to end of study.

### Training

From the 18 CF clinics, 13 clinics had healthcare professionals participate in training. Twelve clinics had healthcare professionals (15 nurses, 1 pharmacist) attend the training workshop in November 2010 and two clinics had healthcare professionals (2 nurses) complete the online tutorial. Fifteen healthcare professionals completed the knowledge test post workshop and two completed the knowledge test post tutorial. Their median knowledge score was 7 out of 10 (mean 6.9; 1.85 SD). Twelve workshop participants completed the satisfaction survey indicating that the workshop met the objectives (*n* = 11–12 out of 12) by providing just the right amount of information (*n* = 10) and role play exercises (*n* = 11), with new information about decision support (*n* = 12). The overall rating of the workshop was excellent or good (*n* = 9; *n* = 3). In the open satisfaction survey questions, workshop participants indicated that they would determine who was ready for transplant discussions, inform team members of who should be targeted, use the patient decision aid, and make sure to also consider patient values.

### Conference calls

Twelve CF clinics had nurse coordinators participate in one or more conference calls with the principle investigators of the study (SA, KV, DS). For each conference call, 6–12 nurses participated and they varied across calls according to who was available when the call was scheduled. Nurses described the decision-making process as the CF physician typically introduced the topic of lung transplant referral and the CF nurse coordinator provided and discussed the patient decision aid with the patients. At least one site discussed having filed the patient decision aid one-page summary report on the patients’ clinic chart for future reference. Nurses also discussed the sensitivities about timing for introducing the patient decision aid. For example, if patients were feeling positive about their ability to manage their CF despite poor lung function, nurses reported that the CF team held off on introducing the patient decision aid at that clinic visit. Several nurses shared the advantages of using the patient decision aid with patients during a hospital admission. For example, hospitalized patients were described as sicker and more likely to be reflecting upon their disease progression. As well, nurses stated that there was time to have longer discussions with patients who were hospitalized and it was easier to have follow-up discussions within 1–2 days of introducing the patient decision aid in hospital.

### Sustained use of the patient decision aid

Of the 18 participating clinics, 15 responded to the baseline patient decision aid use survey and provided tracking logs for the 2 years of the study and three participating clinics did not provide tracking logs. At baseline, the 15 CF clinics reported that the patient decision aid was used by 18 of 62 CF patients (29%) who were considering referral for lung transplantation within the previous year. After initiating our implementation interventions, the 15 CF clinics reported on tracking logs that the patient decision aid was used by 58 of 68 CF patients (85%) considering lung transplantation referral during the first year of the study and 54 of 59 (92%) during the second year of the study (Figure 1; Table 5). There was a statistically significant increase in uptake in the first year (*p* < 0.001), and this uptake was sustained in the second year (see Figure 1).

**Table 5:** **Patient decision aid use during the 2-year study period**

**Site**	**Proportion of patients who received the CF patient decision aid according to the tracking logs**	
	**Year 1**	**Year 2**
1	12/12 (100%)	4/4 (100%)
2	11/13 (85%)	5/5 (100%)
3	8/9 (89%)	4/4 (100%)
4	No patients referred	3/3 (100%)
5	0/1 (0%)	0/4 (0%)
6	1/1 (100%)	No patients referred
7	0/5 (0%)	12/12 (100%)
8	6/7 (86%)	5/5 (100%)
9	3/3 (100%)	4/4 (100%)
10	No patients referred	No patients referred
11	9/9 (100%)	3/4 (75%)
12	3/3 (100%)	2/2 (100%)
13	2/2 (100%)	1/1 (100%)
14	No patients referred	10/10 (100%)
15	3/3 (100%)	1/1 (100%)
*Total*	*58/68* (*85%*)	*54/59* (*92%*)

### Change in perceived barriers to using patient decision aids

Twenty-eight of 31 healthcare professionals completed the survey at baseline and the end of the study (90%), and three completed the survey at baseline only (10%). Tables 3 and 4 show the changes in perceived barriers to using patient decision aids. Significant changes in barriers at the end of the study compared to baseline were: more healthcare professionals felt confident in their ability to support patients making decisions (84% pre to 96% post; *p* = 0.03) but were more likely to need to enhance their ability to support patients handling conflicting views about the decision (71% pre to 50% post; *p* = 0.01). Other remaining barriers were: the need to further enhance their knowledge (55% pre to 50% post) and skills (58% pre to 43% post) in supporting patients making health decisions and perceived lack of time (48% pre to 50% post). Although most (>88%) thought the patient decision aid would be easy to use without requiring re-organization of the CF clinic, only two-thirds thought it was likely to be used by most of their colleagues.

## Discussion

Our pan-Canadian study systematically evaluated the implementation of a patient decision aid in clinical practice. There was good across-Canada participation with 78% of CF clinics involved and an additional 13% that provided data on the patient decision aid use without participating in the implementation interventions. Our study demonstrated that implementation interventions tailored to overcome identified barriers helped us reach our goal of ensuring regular and sustained use of the patient decision aid for 80% or more of adults with CF considering referral for lung transplantation. In fact, there was sustained use of the patient decision aid with over 90% of eligible patients using it during the second year for 15 CF clinics that provided tracking logs.

Our implementation intervention used training to enhance healthcare professional knowledge and skills in using patient decision aids. At the end of the study, more healthcare professionals reported that they felt confident in their ability to support patients making health decisions. However, some participants continued to identify the need to further enhance their knowledge and skills. The median knowledge score of 70% is consistent with trials of healthcare professionals but lower than those completing the test as part of a university-based training program [29]. Our findings are similar to other studies describing the challenges of implementation and the difficulty determining what interventions and intensity of interventions are actually required [13,23]. The differences between our study and previous randomized controlled trials was that healthcare professionals’ performance in our study was not objectively measured using simulated patients, and we added use of conference calls to provide ongoing support beyond the educational workshop [21,28]. These conference calls are similar to reinforcement sessions shown to be effective in other studies focused on implementing shared decision-making [23]. During calls, nurses discussed how the patient decision aid was being used within the CF clinic, ways of introducing the patient decision aid to the patient as part of the process of care, and the timing of follow-up patient discussions. Sharing their experiences provided ideas on how to facilitate patient decision aid use and how to better support patients making this decision.

Our findings appear to support the use of the Knowledge-to-Action Framework. According to this framework, patient decision aids are third-generation knowledge translation tools aimed at presenting second-generation knowledge in user-friendly implementable formats [30]. Second-generation knowledge uses synthesis to aggregate first-generation knowledge of individual studies. The Knowledge-to-Action Framework hypothesizes that implementation is more successful when using a systematic process that includes adapting patient decision aids to the local context and tailoring implementation interventions to overcome known barriers. Healthcare professionals participating in our study identified barriers to using the patient decision aid that appeared to be addressed by the interventions. Another key assumption of this framework is that planning for sustainability is initiated when implementation interventions are being chosen [31]. However, remaining barriers identified at the end of the study require additional interventions to support ongoing implementation.

Despite improved use of the patient decision aids with 15 participating CF clinics, the other 8 CF clinics in Canada that did not fully participate in the study had no or unclear use of the patient decision aid. Therefore, the tailored interventions were not adequate for all eligible CF clinics and qualitative research could be helpful for exploring reasons for non-participation and/or non-use of the patient decision aid. Other countries have policy level interventions to facilitate implementation of patient decision aids. For example, in the United States, there is legislation requiring use of patient decision aids for elective surgical procedures and this would include lung transplantation [32]. The National Health Service in the United Kingdom has a large initiative to implement patient decision aids to enhance shared decision-making across a wide range of health conditions [http://sdm.rightcare.nhs.uk/]. However, there has been no evaluation of the influence of these new health policy-driven initiatives on uptake of patient decision aids.

There are four key limitations of our study. First, there is the possibility of reporting bias. We had no mechanism to independently verify the information that was submitted in the tracking logs by the nurse coordinators. Our original intent was to survey the adult CF patients in each clinic after the first and second year of the study; however, this was not feasible because of privacy and ethical issues. Second, we were unable to engage 100% of CF clinic in Canada. Our intervention appeared to be successful in clinics that fully participated in the study, but some clinics that did not participate in the interventions reported no use of the patient decision aids. Further research should be conducted to explore why some clinics provided data but chose not to participate in the interventions and why other clinics did not participate. Third, use of English language primarily may have limited recruitment and delivery of the study interventions. Although our patient decision aid, study measures, and online tutorial were available in French, the face-to-face workshop and conference calls were in English only. Future implementation studies designed to reach sites across Canada need to ensure all implementation interventions are available in both official languages. Finally, the interventions mostly targeted healthcare professionals and it would be helpful to better monitor the influence of organizational level factors and patients’ responses.

## Conclusion

Despite having presented and published our previous work demonstrating that the CF lung transplant referral patient decision aid improved quality of decisions made by patients, these forms of passive dissemination were inadequate for ensuring uptake of this patient decision aid in routine clinical practice. Our findings suggest the value of systematically investigating the process of implementation of patient decision aids with CF healthcare professionals in order to identify and address barriers perceived to interfere with using the patient decision aid. Our findings indicate that providing tailored interventions to specifically target the modifiable barriers appeared to result in more healthcare professionals feeling confident in their ability to support patients making health decisions and uptake of the patient decision aid within the participating Canadian CF clinics. Our findings also appeared to show sustained use of the patient decision aid during the second year of the study. Further evaluation is required to better understand barriers influencing routine use of patient decision aids in clinical settings resistant to participate.

## Abbreviation

CF: cystic fibrosis
